# Upper gastrointestinal endoscopy; A study from a rural population of Sindh, Pakistan

**DOI:** 10.12669/pjms.37.1.3297

**Published:** 2021

**Authors:** Muhammad Kamran, Asher Fawwad, Syed Iftikhar Haider, Tahir Hussain, Jameel Ahmed

**Affiliations:** 1 Dr. Muhammad Kamran, FCPS. Department of Medicine, Fatima hospital, Baqai Medical University, Karachi, Pakistan; 2 Dr. Asher Fawwad, (PhD). Research Director (Honorary), Research Department of Baqai Institute of Diabetology and Endocrinology, Department of Biochemistry, Baqai Medical University, Karachi, Pakistan; 3 Dr. Syed Iftikhar Haider, MD, Department of Medicine, Fatima hospital, Baqai Medical University, Karachi, Pakistan; 4 Dr. Tahir Hussain, FRCP. Department of Medicine, Fatima hospital, Baqai Medical University, Karachi, Pakistan; 5 Dr. Jameel Ahmed, FRCP. Department of Medicine, Fatima hospital, Baqai Medical University, Karachi, Pakistan

**Keywords:** Gastritis, Hiatal hernia, Indications, Findings

## Abstract

**Objective:**

To discuss common indications and findings on upper gastrointestinal endoscopy as well as to correlate these findings with alarm symptoms in the rural population of Gadap town, Sindh.

**Methods:**

This was a retrospective study on 1288 patients conducted in the medical ward of Fatima Hospital, Baqai Medical University. Patients’ demographics and other data related to the procedure were recovered from patients’ records. SPSS version 20 was used for statistical analysis.

**Results:**

Ratio of male and female patients was approximately 1:1. Majority of the patients were young, and most procedures were done as outpatients without the requirement of conscious sedation. Epigastric pain was the primary indication for upper GI endoscopy (62.6%). One third of the procedures performed did not report any pathological finding. Probability of a positive finding was more likely if a patient presented with dysphagia, heart-burn, hematemesis, vomiting, or for screening endoscopy (for varices). Patients who were diagnosed with esophageal candidiasis, esophageal varices or esophageal growth/ ulcer had reported one or more alarm symptoms in their history.

**Conclusions:**

Upper gastrointestinal endoscopy is a useful test to diagnose disorders of the esophagus, stomach and duodenum. However, it is an expensive procedure and therefore referring physicians should keep appropriate clinical indication and ethical considerations in mind before recommending such an investigation to their patients.

## INTRODUCTION

The prevalence of upper gastrointestinal (GI) tract disorders is enormous in the population in general, both in the developed and the under-developed countries. These disorders are associated with substantial morbidity, and hence the high burden of upper GI tract diseases lead to impairment in the overall quality of life, along with the considerably high healthcare costs related to it.^1^,^2^ Worldwide, millions of people are suffering from dyspepsia, peptic ulcer disease (PUD), gastro esophageal reflux disease (GERD) as well as malignancies of the upper GI tract.^3^ The symptomatology of most of these disorders is based on many factors, including dietary patterns, life-style, Helicobacter pylori infection, and even psychological factors like stressful events in life.^4^ Therefore, it is imperative that these disorders be diagnosed timely and treated appropriately in order to improve the patient’s health related quality of life.

Various imaging modalities are utilized in the work-up of defining the etiology of upper GI complaints. Upper GI endoscopy (UGIE) plays a pivotal role in the diagnosis of these upper GI conditions as it facilitates the operator (endoscopist) to visualize, and carry out biopsies of doubtful lesions with ease and in a cost-effective manner.^5^ UGIE not only aids in reaching a definitive diagnosis in such cases, but also serves as a therapeutic modality in certain emergent situations like GI bleeding, hence enabling the endoscopist to intervene at various phases of the disease process. The procedure is not difficult to perform, and is being carried out not only by fully trained gastroenterologists, but also by internists, and in some cases, general surgeons. Its utilization in picking up and treating gastro-luminal pathologies has evolved extensively over the years. The diagnostic yield of UGIE is better than radiological studies, chiefly in the investigation of upper GI bleeding and various inflammatory disorders of the esophagus, stomach and duodenum as well as certain vascular anomalies.^6^

Nowadays, due to its easy availability, many patients are being recommended UGIE for further evaluation of their symptoms of the upper GI tract, so that sinister pathologies like malignancies can be detected and treated at an early stage. However, this procedure is expensive and still out of reach of many patients, who genuinely require it but cannot afford its cost. Ample data, both national and international, are available with regards to common indications and findings of UGIE.^7^-^10^

Gadap town is situated in the northern part of Karachi city, its population being composed of multi-ethnic groups of mostly poor socio-economic status. As per the national census of 2017, the population of Gadap town is approximately 65,000. The spectrum of diseases found on UGIE in the local populace of Gadap is largely unknown. Therefore, the aim of this study was to describe the key indications and present the common findings seen on upper GI examination among this particular group of patients. Additionally, we also looked at the association of alarm symptoms with the various anomalous findings on UGIE.

## METHODS

This was a retrospective review of the records of all patients who underwent upper GI endoscopy in the medical ward of Fatima Hospital, Baqai Medical University, from December 2016 to May 2019. The study was approved by the Ethics committee (Ref: BMU-EC/07-2019-03, Dated: July 19th 2019) of Baqai Medical University. The gastroscope used was forward viewing endoscope (Olympus Q140 series video endoscope). All procedures were performed after an overnight fast and in the standard manner after obtaining written informed consent. We used 4% xylocaine as the chief form of pharyngeal analgesia, while midazolam as an intravenous sedating agent was mainly reserved for patients who required intervention e.g. endoscopic variceal band ligation (EVBL). The endoscopic procedures were performed in patients who were either already admitted in wards, or as an out-patient setting (either referrals from the out-patients department of Fatima Hospital or from private clinics within close proximity of the hospital). After the procedure, patients were observed for approximately one hour for possible post-procedure complications, before either being discharged home or shifted to their respective wards.

Data regarding age and gender of the patients, their ethnicity, body mass index (BMI), procedure setting (either inpatient or outpatient), primary indication of the procedure, endoscopic findings and final diagnosis was retrieved from the records. Abnormal endoscopy was considered as one in which there were macroscopic luminal findings. Biopsies were routinely taken from the lesions for histopathological examination.

Data entry and analysis was performed using statistical package for social sciences (SPSS) version 20. Descriptive variables were delineated as frequencies and percentages. Presence of upper GI bleeding (either in the form of hematemesis or melena), dysphagia, vomiting, weight loss and/ or iron deficiency anemia were included as alarm symptoms. The probability of picking up a positive finding on UGIE was expressed as odds ratios (O.R) with 95% confidence intervals (C.I), as was the relationship between different alarm symptoms and respective findings based on UGIE.

## RESULTS

The characteristics of the study population are summarized in Table-I. A total of 1288 patients went through a complete upper GI endoscopic examination over the period. Males and females were almost equally proportionate (50.9% and 49.1% respectively), with slightly higher average BMI values in women as opposed to men (19.9 and 19.5 respectively). Majority of patients belonged to younger age group (20-39 years of age), whereas only 1.2% were over 79 years old. Almost half of the patients were of Pushto ethnicity. Most of the patients underwent the procedure as outpatients (82.6%), with pharyngeal analgesia being used as the sole agent of analgesia in the majority (96.7%). The primary indication as well as the major findings on UGIE are shown in Table-II. The most common reason for patients to undergo UGIE was found to be epigastric pain (62.6%). Other noteworthy primary indications were heart-burn, screening for varices in patients with chronic liver disease, iron deficiency anemia and vomiting. As far as the endoscopic findings were concerned, approximately one third of the patients (35.4%) had a macroscopically normal examination. However, prominent gross pathologic findings included gastritis, hiatal hernia, presence of esophageal/ gastric varices and esophagitis (both candida and non-candida associated). The correlation between specific procedure indication and endoscopic finding are shown in Table-III. Presence of dysphagia, heart-burn, hematemesis, performing a screening endoscopy (for varices) and vomiting all were significantly associated with a positive pathological finding on UGIE.

**Table-I:** Patient characteristics.

Variables	Number of patients (%)
***Age (years)***	
<20	77 (6.0%)
20-39	649 (50.4%)
40-59	408 (31.7%)
60-79	139 (10.8%)
>79	15 (1.2%)
***Gender***	
Male	656 (50.9%)
Female	632 (49.1%)
Ethnicity	
Sindhi	495 (38.4%)
Pushto	640 (49.7%)
Urdu speaking	109 (8.5%)
Punjabi	17 (1.3%)
Balochi	23 (1.8%)
Brohi	4 (0.3%)
***BMI (average)***	
Male	19.5
Female	19.9
***Procedure setting***	
Out-patient	1064 (82.6%)
In-patient	224 (17.4%)
***Conscious sedation***	
Yes	42 (3.3%)
No	1246 (96.7%)

**Table-II:** Primary indications and Predominant findings on upper GI endoscopy.

Indications	Number of patients (%)	Endoscopic findings	Number of patients (%)
Epigastric pain	806 (62.6%)	Normal examination	456 (35.4%)
Vomiting	39 (3.0%)	Gastritis	415 (32.2%)
Heart-burn	208 (16.1%)	Esophageal candidiasis	8 (0.6%)
Dysphagia	31 (2.4%)	Non-candida esophagitis (including Barrett’s)	29 (2.3%)
Screening EGD (CLD)	93 (7.2%)	Hiatal hernia	174 (13.5%)
Iron deficiency anemia	45 (3.5%)	Patulous GE junction	14 (1.1%)
Chronic diarrhea	5 (0.4%)	Duodenal fissuring	27 (2.1%)
Weight loss	18 (1.4%)	Esophageal/ gastric varices	102 (7.9%)
Hematemesis	28 (2.2%)	Esophageal growth	22 (1.7%)
Melena	10 (0.8%)	Esophageal ulcer	4 (0.3%)
Hiccups	4 (0.3%)	Gastric growth	10 (0.8%)
Caustic ingestion	1 (0.1%)	Gastric ulcer	8 (0.6%)
		Duodenal growth	5 (0.4%)
		Esophageal/ duodenal diverticulae	6 (0.5%)
		Achalasia	2 (0.2%)
		Scleroderma esophagus	1 (0.1%)
		Worm infestation	3 (0.2%)
		Food bolus impaction in esophagus	1 (0.1%)

**Table-III:** Relationship between procedure indication and an abnormal positive endoscopic finding.

Indication for endoscopy	Normal finding	Positive finding	OR(95% C.I)	P-value
n	456	832		
Epigastric pain	323(70.8%)	483(58.1%)	1	
Dysphagia	7(1.5%)	24(2.9%)	3.43(1.48-7.96)	0.004
Heart-burn	67(14.7%)	141(16.9%)	2.1(1.57-2.82)	<0.0001
Hematemesis	7(1.5%)	21(2.5%)	3(1.28-7.06)	0.012
Hiccups	0(0%)	4(0.5%)	-	-
Iron deficiency anemia	20(4.4%)	25(3%)	1.25(0.69-2.25)	0.457
Melena	2(0.4%)	8(1%)	4(0.85-18.84)	0.08
Screening EGD (CLD)	10(2.2%)	78(9.4%)	7.8(4.04-15.07)	<0.0001
Vomiting	12(2.6%)	27(3.2%)	2.25(1.14-4.44)	0.019
Weight loss	5(1.1%)	13(1.6%)	2.6(0.93-7.29)	0.069
Surveillance EGD (CLD)	0(0%)	5(0.6%)	-	-
Chronic diarrhea	3(0.7%)	2(0.2%)	0.67(0.11-3.99)	0.657
Caustic ingestion	0(0%)	1(0.1%)	-	-

Reference category for odd ratio (OR): Epigastric pain, P-value<0.05 considered to be statistically significant.

The O.R’s of endoscopically identifying different conditions in patients presenting with alarm symptoms, as opposed to those without alarm symptoms, are presented in Table-IV. Here, we found that although patients with findings of gastritis and hiatal hernia presented without alarm symptoms (significant p-value in both cases), those with esophageal candidiasis, esophageal varices or esophageal growth/ ulcer diagnosed on UGIE had one or more alarm symptoms to begin with.

**Table-IV:** Findings of upper GI endoscopy in patients with and without alarm symptoms.

Endoscopic Finding	Non-alarm symptoms	Alarm symptoms	OR (95% C.I)	P-value
n	1117	171	-	-
Normal examination	402(36%)	54(31.6%)	1	
Gastritis	401(35.9%)	15(8.8%)	0.04(0.02-0.06)	<0.0001
Esophageal candidiasis	5(0.4%)	3(1.8%)	0.6(0.14-2.51)	<0.0001
Non-candida esophagitis including Barrett’s	24(2.1%)	5(2.9%)	0.21(0.08-0.55)	0.484
Hiatal hernia	157(14.1%)	18(10.5%)	0.12(0.07-0.19)	0.001
Patulous GE junction	14(1.3%)	0(0%)	-	
Duodenal fissuring	7(0.6%)	20(11.7%)	2.86(1.21-6.76)	0.998
Esophageal varices	85(7.6%)	17(9.9%)	0.2(0.12-0.34)	0.017
Esophageal growth	3(0.3%)	18(10.5%)	6(1.77-20.37)	<0.0001
Esophageal ulcer	3(0.3%)	1(0.6%)	0.33(0.04-3.21)	0.004
Gastric growth	4(0.4%)	7(4.1%)	1.75(0.51-5.98)	0.341
Gastric ulcer	2(0.2%)	6(3.5%)	3(0.61-14.86)	0.372
Duodenal growth	3(0.3%)	2(1.2%)	0.67(0.11-3.99)	0.178
Esophageal/duodenal diverticulae	3(0.3%)	3(1.8%)	1(0.2-4.96)	0.657
Achalasia	1(0.1%)	1(0.6%)	1(0.06-15.99)	0.999
Worm infestation	2(0.2%)	1(0.6%)	0.5(0.05-5.51)	0.999
Food bolus impaction in esophagus	1(0.1%)	0(0%)	-	

Reference category for odd ratio (OR): Normal examination, P-value<0.05 considered to be statistically significant.

## DISCUSSION

Our study was a retrospective record of upper GI endoscopy of nearly thirteen hundred patients of Gadap town and its periphery. Approximately half of the subjects undergoing upper GI endoscopy were young (i.e. under 40 years of age). This was contrary to the results from a similar studies conducted few years ago, where majority of the patients were in their 4^th^ decade of life or beyond.^11^,^12^ However, a recent study from Bangladesh reported a high prevalence of dyspepsia in people < 40 years.^13^ This may be true for our younger population as well, hence the need of further investigations including endoscopic procedures. Male to female ratio in our study was found to be approximately 1:1, comparable to previous works.^11^

We performed most of the diagnostic endoscopic procedures without conscious sedation, using pharyngeal throat spray alone. This mode of analgesia was not only well-tolerated by majority of the patients, but was also cost-effective. A local study also demonstrated minimal risk of hypoxia in patients undergoing upper GI endoscopy without conscious sedation.^14^ Although upper GI endoscopy is a relatively risk-free technique, there may be occasional complications like luminal perforation, bleeding in the GI tract, aspiration pneumonitis, cerebro-vascular accident and super-imposed infections. Fortunately, we did not find any record of immediate post-procedural complications from the discharge notes of our patient cohort. However, we are not aware of any delayed complications if any, since they were not mentioned in the patients’ records.

Nearly two-thirds of our patient population underwent upper GI endoscopy because of long-standing epigastric pain. This is a very common complaint among patients suffering from dyspepsia, and has been the most frequent reason for UGIE in previous studies as well.^6^,^8^ The second most common indication highlighted in this study was heart-burn followed by screening endoscopy for the presence of esophageal or gastric varices (in cases of chronic liver disease).

Identification of organic disease (particularly malignancy), is usually the main reason for endoscopy. Therefore, endoscopic quality is affected by inappropriate procedure indications, not to forget the financial and psychological burden on patients.^15^ The guidelines from the American Society for Gastrointestinal Endoscopy (ASGE) also recommend endoscopy for “high risk” patients, including patients over age 50 years with new-onset dyspepsia, presence of alarm symptoms (dysphagia, weight loss, evidence of GI bleeding, vomiting).^16^ From our retrospective review, we learnt that approximately one third of the subjects undergoing UGIE in our hospital had normal endoscopic finding. This, although a high number, is not an unexpected observation as a number of earlier studies have also reported the same.^8^,^12^,^15^

In our scenario, the reason for not finding a significant abnormality in a substantial number of procedures may be the fact that most cases were referrals from physicians and general practitioners from private clinics in the hospital vicinity. Hence the predominant indication for upper GI endoscopy (whether appropriate or otherwise) was ascertained by the doctor primarily looking after the patient, with the endoscopists performing the procedure as indicated in the request form. As a result, the endoscopists lacked key information regarding some of the patients’ history, clinical examination and other investigative data, putting the appropriateness of the procedure indication in question.

This said, it has been seen based upon individual experiences that even incorrect procedure indications may sometimes be valuable from the patient’s standpoint as it is a source of reassurance and satisfaction to him, and also improves his quality of life by reducing the fear of a serious diagnosis like cancer (in the presence of normal endoscopic findings). Additionally, evidence has shown that this phenomenon also results in decreased number of physician consultations as well as poly-pharmacy.^17^,^18^ However, it would remain inappropriate and unethical to carry out costly endoscopic procedures without suitable indication. Clinical judgment should be part of this decision making process, although at times it may possibly be considered incongruous based on current ASGE guidelines. At the same time, sincere efforts should be made in the form of educational and other training programs for referring physicians in order to utilize the endoscopic facility in a clinically beneficial and cost-effective manner.^19^

In this regard, our results clearly show that the probability of detecting a pathological finding on UGIE is more if the patient is giving an obvious history of dysphagia, hematemesis or heart-burn, or is undergoing a screening endoscopy for varices. Additionally, we also elucidated that esophageal cancers, varices and candidiasis were more likely to be found on UGIE in patients with one or more alarm symptoms with regards to the upper GI tract, whereas patients presenting without alarm symptoms mostly revealed findings of gastritis and hiatal hernia.

The main strength of our study was the clinical data presentation of a substantial number of patients with upper GI tract disorders and diverse ethnic backgrounds, which, to the best of our knowledge, has never been performed in this population cohort.

### Limitations of the study

Firstly, this was data from a single center, and the results may not be the true representative of the whole population in question. This said, Gadap town predominantly comprises financially deprived inhabitants, and our hospital, being a charitable organization, naturally serves the majority of patient population in this area. Secondly, with retrospective designs, proper causative extrapolations among the study variables could not be drawn. Thirdly, we did not perform routine biopsies in every patient. Only macroscopically abnormal lesions were sampled and sent for histopathological analysis. This was because, although apparently normal GI mucosa may harbor microscopic disease, biopsies incur an additional cost which, in a resource-constraint society like ours, may put an extra financial burden on the patient.^20^

## CONCLUSIONS

Upper GI endoscopy is a crucial invasive investigation to recognize precise pathologies in patients who present with symptoms of the upper GI tract. This single large retrospective analysis demonstrated epigastric pain to be the most frequent indication, and normal endoscopic finding to be the commonest outcome of an upper GI endoscopic procedure. For the under-privileged residents of Gadap town, upper GI endoscopy is still a costly procedure and thus difficult for patients to access. Therefore, appropriateness of clinical indication is of the essence before carrying out such an expensive investigation in order to optimize resource utilization.

### Author’s Contribution:

**KM:** conceived, designed and performed manuscript writing. **HIS:** helped in data collection edited and final approval of manuscript. **FA:** did statistical analysis and data interpretation final approval of manuscript. **AJ, HT & HIS:** did review and final approval of manuscript.
